# Supplementary material for: Construction of Direct Z−Scheme SnS2 Quantum Dots/Conjugated Polyimide with Superior Photocarrier Separation for Enhanced Photocatalytic Performances
Source: Polymers (Basel). 2022 Dec 14;14(24):5483. doi: 10.3390/polym14245483 (PMC9782883; doi:10.3390/polym14245483)
Supplement: Supplementary file 1 [file polymers-14-05483-s001.zip › polymers-2024420-supplementary.pdf]

Construction of direct Z−scheme SnS_2 quantum dots/conjugated polyimide with superior photocarrier separation for enhanced photocatalytic performances

Changqing Yang, Chenghai Ma,* Duoping Zhang, Zhiang Luo, Meitong Zhu, Binhao Li, Yuanyuan Zhang, Jiawei Wang

School of Chemical Engineering, Qinghai University, Xining 810016, China

* Corresponding author at: School of Chemical Engineering, Qinghai University, Xining, 810016, PR China.
E-mail address:chmaqhu@126.com (C. Ma).

Table S1. The amounts of the reactants for synthesis of the SQDs/SPI samples

Samples*	Reactants (g)			
	SPI	$SnCL_4.5H_2O$	C_2H_5NS	Synthetic yield
1SQDs/SPI	0.990	0.0192	0.0166	0.9965
3SQDs/SPI	0.970	0.0576	0.0494	0.9896
5SQDs/SPI	0.950	0.0959	0.0822	0.9953
10SQDs/SPI	0.900	0.1918	0.1644	0.9682
13SQDs/SPI	0.870	0.2493	0.2137	0.9745
15SQDs/SPI	0.850	0.2876	0.2465	0.9624

*All samples were synthesized by a facile immersion-hydrothermal method, heating at 140 ℃ for 4 hours. The amounts of the reactants were calculated according to the synthesis of 1 g sample.

Table S2. Time-resolved fluorescence decay parameters of SPI, 10SQDs/SPI and SnS_2 samples.

**Samples***	**A_1 (%)**	**τ_1 (ns)**	**A_2 (%)**	**τ_2 (ns)**	**$<\tau_{av}>$(ns)**
**10SQDs/SPI**	**199.41**	**1.50**	**70.21**	**6.49**	**4.51**
**SPI**	**177.23**	**1.79**	**27.59**	**6.48**	**3.48**
**SnS_2**	**117.73**	**1.41**	**14.72**	**9.46**	**1.81**

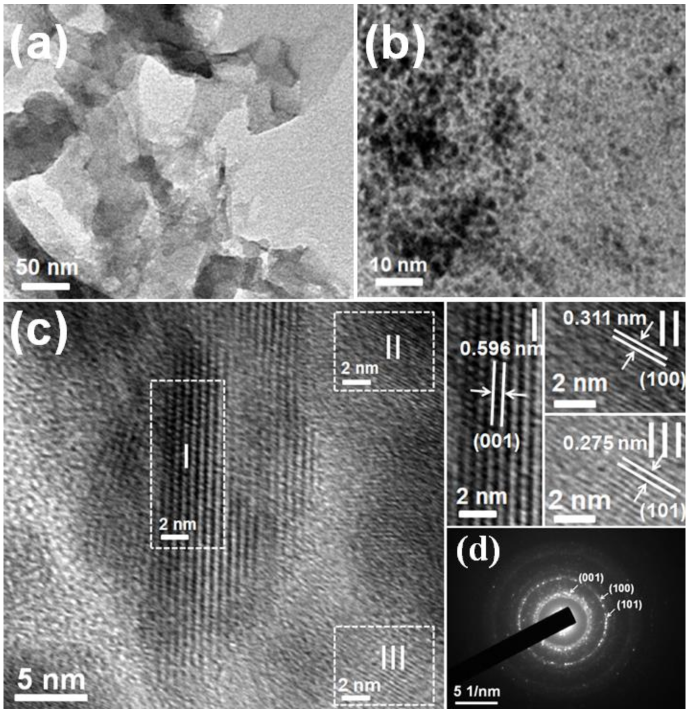

Figure S1. TEM images of (a) SPI and (b) SQDs, (c) HR-TEM, and (d) the SAED image of SQDs sample.

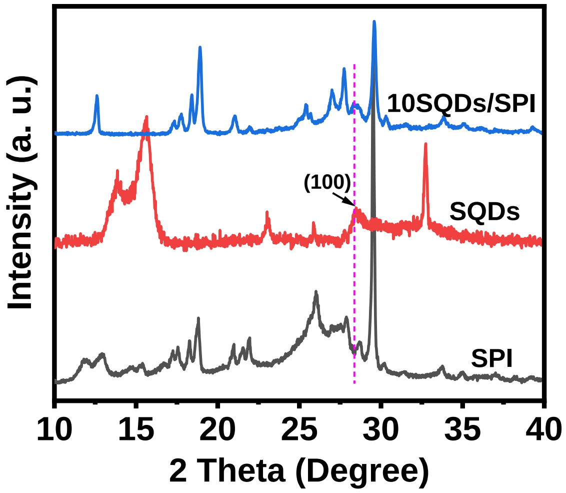

Figure S2. XRD patterns of SPI, SQDs, and 10SQDs/SPI powder samples.

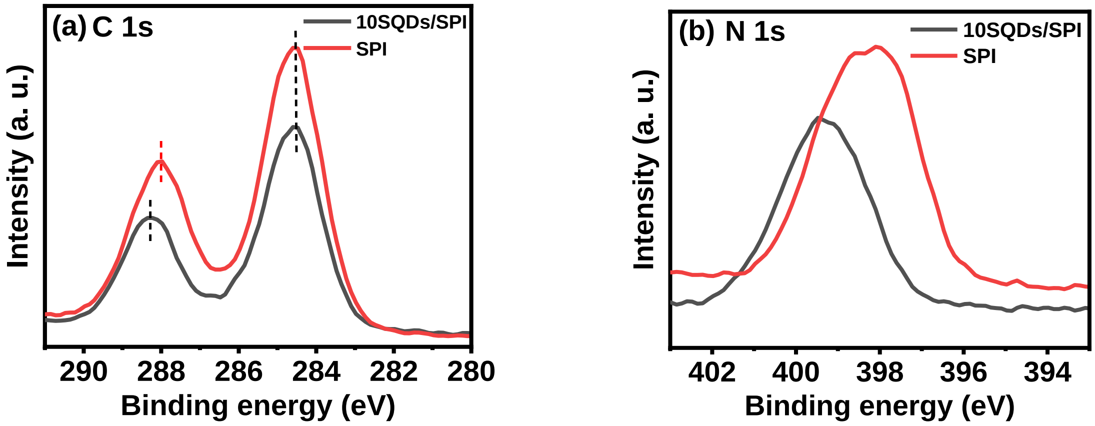

Figure S3. XPS spectra of (a) C 1s, (b) N 1s of SPI and 10SQDs/SPI composite samples.

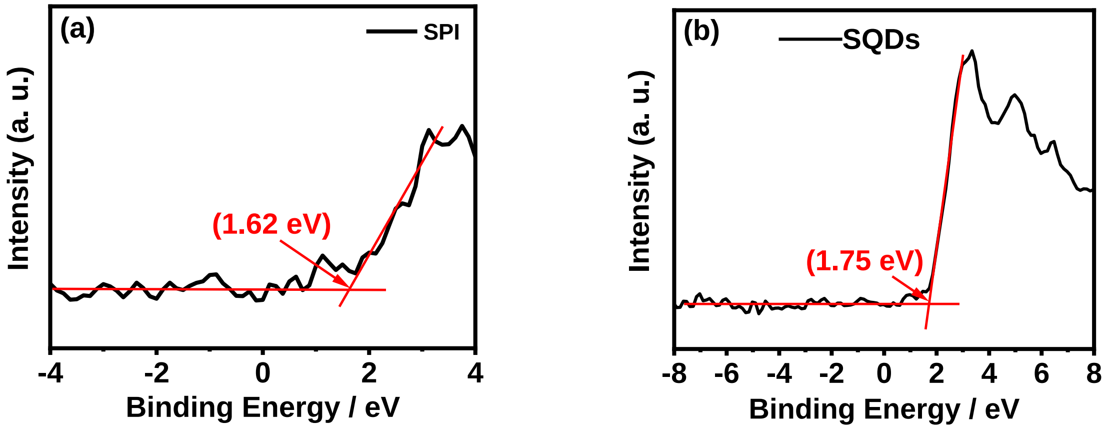

Figure S4. VBXPS spectra of (a) SPI and (b) SQDs samples.

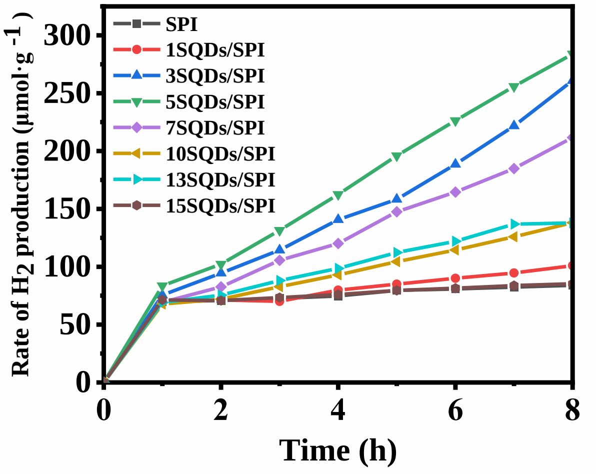

Figure S5. Time course of H_2 evolution of SPI and SQDs/SPI composites loaded 3%Pt under simulates sunlight irradiation.

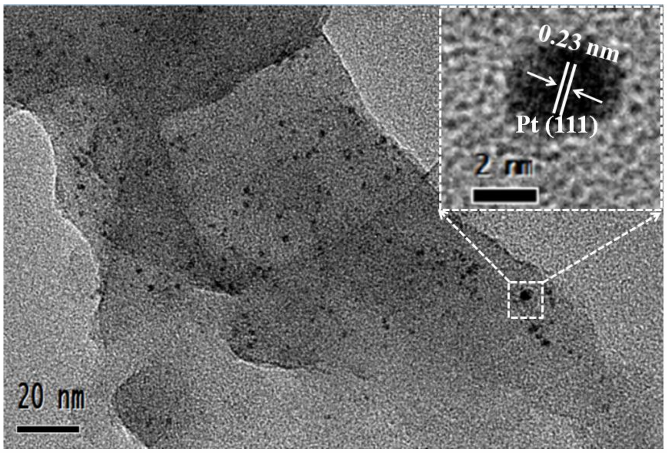

Figure S6. TEM image of 10SQDs/SPI samples loaded with 3% Pt. The inset is a high resolution TEM image.

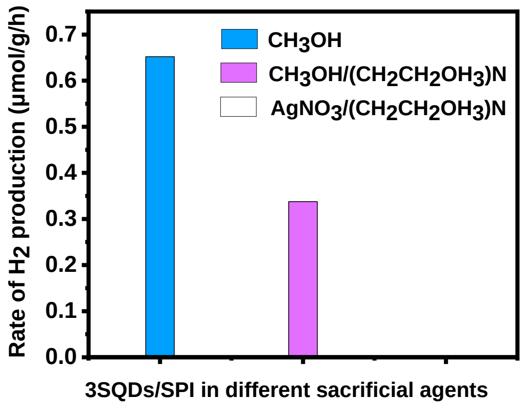

Figure S7. Time course of H_2 evolution of the 3SQDs/SPI composite in anhydrous CH_3OH, anhydrous $CH_3OH/(CH_2CH_2OH)_3N$ and $AgNO_3/(CH_2CH_2OH)_3N$ under light irradiation.

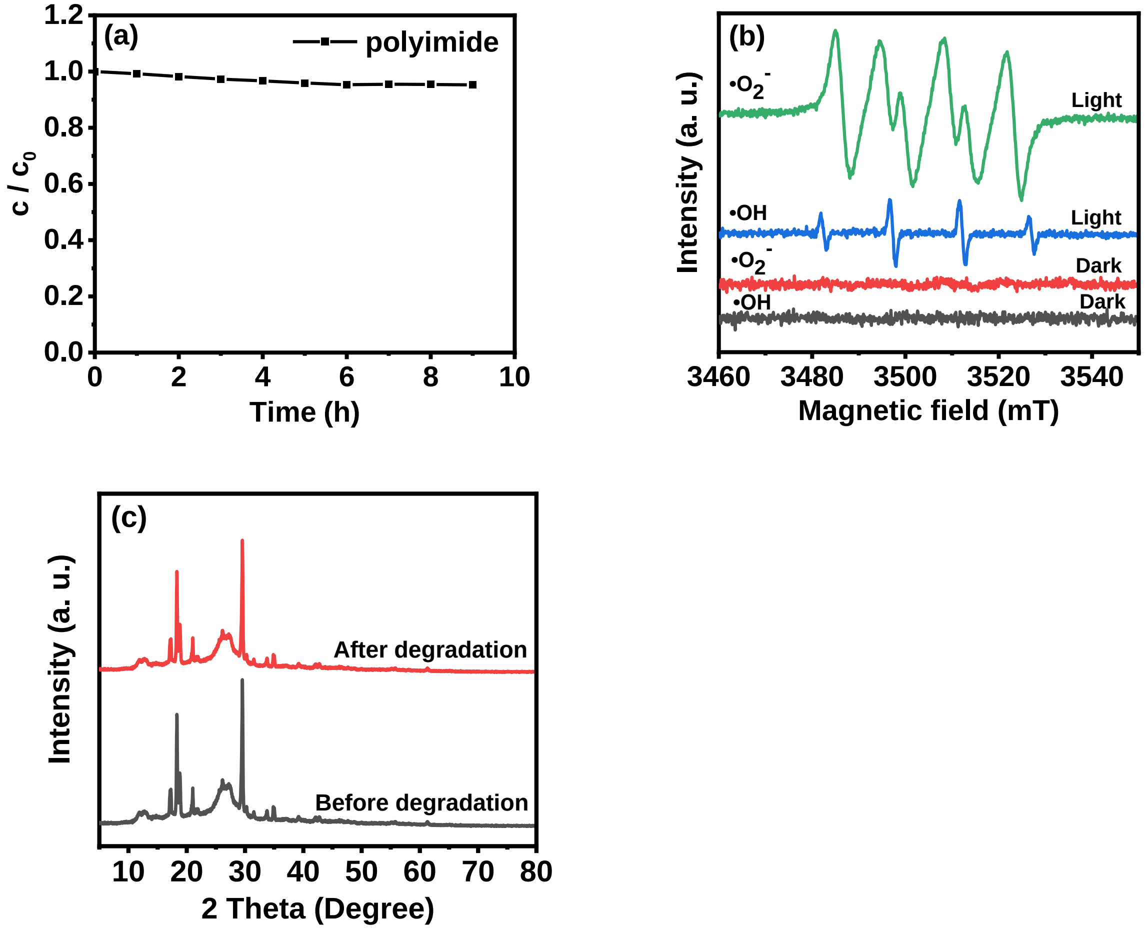

Figure S8. (a) The curve of photodegradable polyimide under full arc light ($\lambda > 300$ nm), (b) DMPO spin-trapping ESR spectra of polyimide in dark and light conditions (in aqueous for DMPO-·OH; in methanol for DMPO-·O_2^-), and (c) XRD patterns of the polyimide before and after degradation.

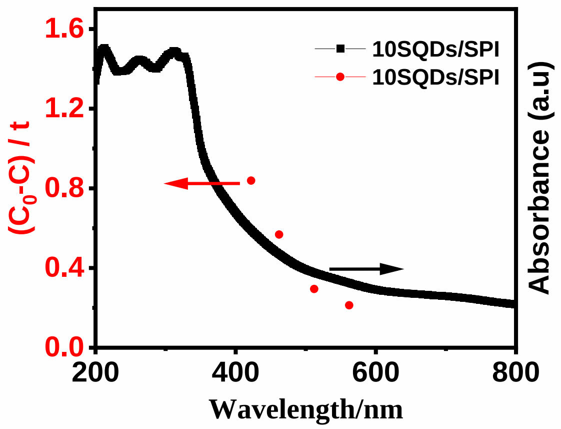

Figure S9. Dependence of degradation activity on wavelength by10SQDs/SPI composite sample.

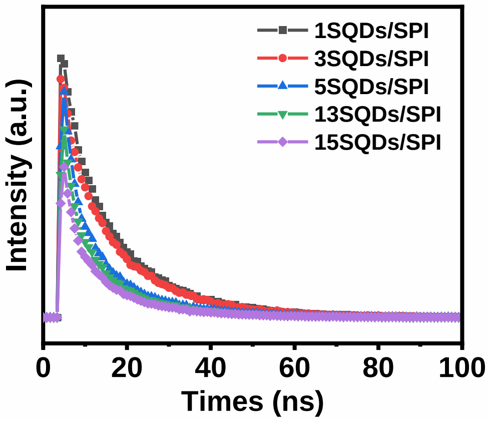

Figure S10. Transient PL decay spectra for 1SQDs/SPI, 3SQDs/SPI, 5SQDs/SPI, 13SQDs/SPI, and 15SQDs/SPI samples.
